# ZNF804A Transcriptional Networks in Differentiating Neurons Derived from Induced Pluripotent Stem Cells of Human Origin

**DOI:** 10.1371/journal.pone.0124597

**Published:** 2015-04-23

**Authors:** Jian Chen, Mingyan Lin, Anastasia Hrabovsky, Erika Pedrosa, Jason Dean, Swati Jain, Deyou Zheng, Herbert M. Lachman

**Affiliations:** 1 Department of Psychiatry and Behavioral Sciences, Albert Einstein College of Medicine, Bronx, New York, United States of America; 2 Department of Genetics, Albert Einstein College of Medicine, Bronx, New York, United States of America; 3 Dominick Purpura Department of Neuroscience, Albert Einstein College of Medicine, Bronx, New York, United States of America; 4 Department of Neurology, Albert Einstein College of Medicine, Bronx, New York, United States of America; 5 Department of Medicine, Albert Einstein College of Medicine, Bronx, New York, United States of America; The George Washington University, UNITED STATES

## Abstract

*ZNF804A* (Zinc Finger Protein 804A) has been identified as a candidate gene for schizophrenia (SZ), autism spectrum disorders (ASD), and bipolar disorder (BD) in replicated genome wide association studies (GWAS) and by copy number variation (CNV) analysis. Although its function has not been well-characterized, ZNF804A contains a C2H2-type zinc-finger domain, suggesting that it has DNA binding properties, and consequently, a role in regulating gene expression. To further explore the role of ZNF804A on gene expression and its downstream targets, we used a gene knockdown (KD) approach to reduce its expression in neural progenitor cells (NPCs) derived from induced pluripotent stem cells (iPSCs). KD was accomplished by RNA interference (RNAi) using lentiviral particles containing shRNAs that target *ZNF804A* mRNA. Stable transduced NPC lines were generated after puromycin selection. A control cell line expressing a random (scrambled) shRNA was also generated. Neuronal differentiation was induced, RNA was harvested after 14 days and transcriptome analysis was carried out using RNA-seq. 1815 genes were found to be differentially expressed at a nominally significant level (p<0.05); 809 decreased in expression in the KD samples, while 1106 increased. Of these, 370 achieved genome wide significance (FDR<0.05); 125 were lower in the KD samples, 245 were higher. Pathway analysis showed that genes involved in interferon-signaling were enriched among those that were down-regulated in the KD samples. Correspondingly, *ZNF804A* KD was found to affect interferon-alpha 2 (IFNA2)-mediated gene expression. The findings suggest that ZNF804A may affect a differentiating neuron’s response to inflammatory cytokines, which is consistent with models of SZ and ASD that support a role for infectious disease, and/or autoimmunity in a subgroup of patients.

## Introduction

*ZNF804A* has been implicated in SZ and BD in replicated genetic and molecular studies [1–2]. In addition, rare copy gain and copy loss CNVs, and an inversion affecting the gene have been found in patients with ASD, psychosis, developmental disabilities, and anxiety disorder [3–6]. *ZNF804A* codes for a protein containing a C2H2-type zinc-finger domain, suggesting that it has DNA binding properties and can act as a transcription factor. This is supported by Girgenti *et al*. who showed that the protein localized to the nucleus, and more specifically to the promoters of several SZ candidate genes in E13 rat embryo cortical neural progenitors [7]. In addition, Hill *et al*. knocked down *ZNF804A* expression in NPCs derived from human cortical neuroepithelium using siRNAs and identified 152 differentially expressed genes by microarray analysis, including genes involved in cell adhesion, neural migration, neurite outgrowth and synaptogenesis [8].

*ZNF804A* is one of many genes coding for transcription factors, splicing regulators and chromatin remodeling proteins that have been implicated in SZ, BD, and ASD, including *POU3F2*, *A2BP1*, *MYT1L*, *JARID2*, *TCF4*, *ARNT2*, *CHD8*, *SMARCA2*, and β-catenin (which is regulated by the lithium and DISC1 target GSK3β) [9–24]. Analyzing the effect of these genes on downstream targets is a reasonable strategy for identifying gene networks—both unique and shared—involved in neuropsychiatric disorders.

To further enhance our understanding of the role of *ZNF804A* on downstream targets, and to expand on the findings reported by Hill *et al*., we have knocked down the gene in NPCs derived from iPSCs with shRNA vectors. Using iPSCs as a source of human neural progenitor cells (NPCs) for genetic manipulation has some advantages over others, such as nasal neuroepithelium and cortical neuroepithelium, one of which is the capacity to generate patient-specific neurons [25–27]. In addition, since iPSCs are self-renewing, permanent, genetically manipulated cell lines can be generated. In this study, we report on a transcriptome analysis carried out on early differentiating human neurons derived from iPSC-generated NPCs in which *ZNF804A* has been knocked down using shRNAs.

## Methods and Materials

### Development of iPSC lines

The study was approved by the Albert Einstein College of Medicine Committee Institutional Review Board (IRB). All participants signed an informed consent that was approved by the Einstein IRB. For this study, *ZNF804A* knockdown (KD) was carried out on a line from a healthy male control. IPSCs were generated using non-integrating plasmids containing *OCT4*, *SOX2*, *KLF4*, *L-MYC*, *LIN28*, and a p53 shRNA vector, introduced by nucleofection [28–31]. The iPSCline used in this experiment has a normal karyotype, expresses pluripotency markers, and is capable of differentiating into all 3 germ layers. S1 Fig and S1 Text).

### Generating NPCs and inducing neuronal differentiation

The protocol for generating NPCs and inducing neuronal differentiation has been previously described [32–34]. Details can be found in the S1 Text **.**

### Lentiviral particle transduction

NPCs were transduced with a mix of three shRNAs targeting *ZNF804A* (Santa Cruz Biotechnology sc-94548-V). In addition, a scrambled shRNA vector was used as a control (Santa Cruz Biotechnology, sc-108080). NPCs were grown in NBF medium (DMEM/F12, 0.5 x N2, 0.5 x B27 and1% P/S) containing FGF2 (20ng/ml) in 6-well plates coated with poly-L-ornithine hydrobromide and laminin. Cells at ~50% confluence were fed with fresh medium and 5ug/ml Polybrene (Santa Cruz Biotechnology, sc-134220) and transduced with shRNA lentiviral particles. Cells were incubated for 24 hours, after which medium with virus was removed and replaced with complete NBF medium without Polybrene. After 24–48 hours, 5 ug/ml puromycin was added to select for transduced cells. Medium with puromycin was changed every other day for 5–7 days. Transduced cells were expanded and induced to differentiate into neurons once ~50% confluence was reached, which occurred approximately 5–10 days after expansion). Cells were harvested for RNA after 14 days of differentiation. Two separate NPC preps from the same KD experiment were induced to differentiate into neurons (scrambled 1, 2; KD 1, 2). In addition, a separate KD was carried out using different shRNA vectors (scrambled 3, KD 3) supplied by the Albert Einstein College of Medicine shRNA Core Facility (S1 Text). The shRNAs contained the following *ZNF804A* target sense sequences: Albert Einstein College of Medicine shRNA core facility (AGAAGCUUAGUUCUUCAAA); Santa Cruz, sc-94548-VA (CAAGGAUGUAUCUACAGAA), sc-94548-VB (GAAGCAGAGAAUAGUUACA), sc-94548-VC (CAACCACCAUUACCAUUCA).

### RNA-seq

Paired-end RNA-seq was carried out on an Illumina HiSeq 2000. We obtained 101-bp mate-paired reads from DNA fragments with an average size of 250-bp (standard deviation for the distribution of inner distances between mate pairs is approximately 100 bp). RNA-seq reads were aligned to the human genome (GRCh37/hg19) using the software TopHat (version 2.0.8) [35]. The category of transcripts used for our expression analysis is described at http://vega.sanger.ac.uk/info/about/gene_and_transcript_types.html. We counted the number of RNA-seq fragments mapped to each gene annotated in the GENCODE database (version 18) [36]. The count was divided by effective gene length (derived from regions covered by reads) to obtain expression level in Transcripts Per Million (TPM), which is calculated by multiplying the estimated fraction of transcripts made up by a given gene by 10^6^ [37]. The measure is independent of the mean expressed transcript length and is thus more comparable across samples; thus it is favored over another popular transcript measure, FPKM, based on previous studies [38]. We used DESeq (an R package developed by Anders and Huber) to determine differential expression from the count data [39]. Afterwards, only genes with average TPMs greater than 1 across samples (n = 15,330) were considered for differential expression. A multiple comparison correction was applied to adjust the p-values for false discovery rate (FDR) [40]. Sequencing data have been deposited in the Gene Expression Omnibus (GEO) repository (accession number GSE54112).

### Quantitative real-time PCR (qPCR)

qPCR was carried out on reverse transcribed PCR using the 2^-ΔΔCt^ method as previously described [30,34]. A detailed description and the primers used for this analysis can be found in S1 Text.

### Proliferation assay

Cell proliferation was assayed using the Vybrant MTT cell proliferation assay kit (invitrogen) according to the protocol manual. Briefly, similar amounts of NPCs (50,000 cells in triplicate) were seeded on 96 well plates coated with poly-L-ornithine hydrobromide and laminin. Cells were assayed daily for 5 days. At the time of the assay, 100ul of medium was removed from the well and replaced with an equal volume of fresh medium without FGF2, along with 10ul of the 12mM MTT (3-[4,5-dimethylthiazol-2-yl]-2,5-diphenyltetrazolium bromide) stock solution. The cells were incubated at 37°C for 4 hours. 85 ul of medium was removed and 50 ul of DMSO was added, followed by a 10 minute incubation at 37°C. The samples were mixed well, transferred to a microplate, and the absorbance at 540 nm was determined.

### Interferon inducible gene expression

NPC cultures were prepared from the parent iPSC line (untreated control), a *ZNF804A* KD and a scrambled shRNA control (KD2 and scrambled 2). The NPCs were previously frozen in aliquots of 10^6^ cells. After thawing, the cells were cultured for several days until 50% confluence was reached, after which they were treated with interferon-alpha 2 (IFNA2; 1mg/ml) or vehicle for 4 hours. IFNA2 was chosen because IPA analysis showed that this was the most significant upstream regulator among the down-regulated genes. RNA was harvested and analyzed by qPCR. Duplicate cultures were treated and analyzed in triplicate for qPCR. We analyzed several IFN-inducible genes, including *IFITM2*, *IFITM3*, *IFI6*, *STAT1* and *STAT3*. However, neither *IFITM2* nor *IFITM3* were significantly affected by IFNA2 treatment under our experimental conditions (not shown). Consequently, the analysis of IFN-inducible genes was restricted to *IFI6*, *STAT1* and *STAT3*. Relative expression values were normalized to β2M expression. The relative expression means were then normalized to a control, vehicle-treated sample, which was arbitrarily set at 1.0. Fold differences were calculated and statistical significance was determined using a Student’s t-test.

## Results

### RNA-seq

**Fig 1:**
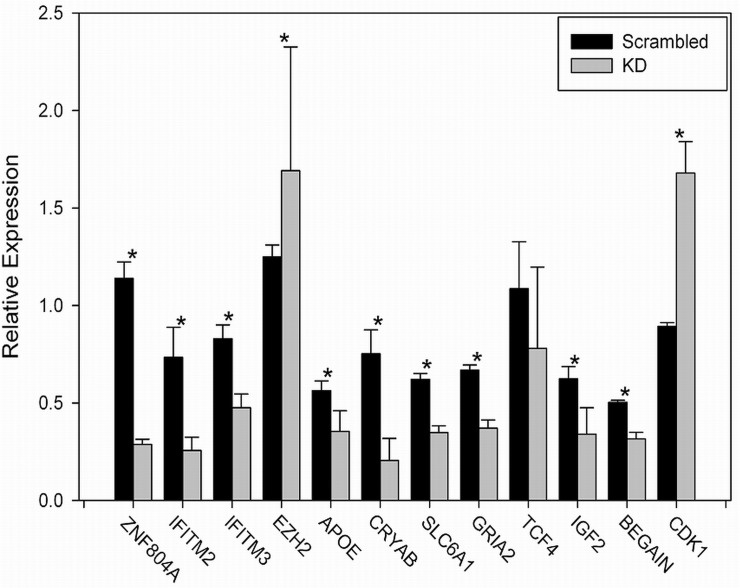
Validation of selected genes by qPCR. qPCR was carried out as described in methods. Each assay was carried out in triplicate, and the mean fold change was calculated. This was repeated 3–6 times for each sample. Asterisk indicates significance at p<0.05 calculated using a Student’s t-test. Actual p-values are: *ZNF804A* (0.003); *IFITM2* (0.040); *IFITM3* (0.001); *EZH2* (0.016); *APOE* (0.018); *CRYAB* (0.015); *SLC6A*1 (0.0004); *GRIA2* (0.011); *TCF4* (0.710); *IGF*2 (0.0004); BEGAIN (0.010); *CDK*1 (0.015).

A total of 1,815 genes were found to be differentially expressed at a nominally statistically significant level (p<0.05); 809 decreased in expression in the KD samples, while 1,106 increased (S1 Table for complete dataset). Of these, 370 reached genome wide significance after correction for multiple testing (FDR<0.05); 125 were lower in the KD samples, 245 were higher. This included 10 long non-coding RNAs that decreased in expression and seven that increased. Reduced expression of *ZNF804* in the KD was confirmed by the RNA-seq findings, which showed a 69% decrease (p = 9.4e-08; FDR = 6.7e-05), as well as by qPCR (S1 Table, Fig 1). The most significantly down-regulated genes based on FDR were *TIMP1*, *LINC00645*, *IFITM3*, *IFITM2*, *KAL1*, *RTL1*, and *PCDH7* (Table 1). *TIMP1* is metallopeptidase inhibitor that has an effect on cellular proliferation, especially in response to cytokines, and acts as an inflammatory mediator in the brain [41]; a connection to immune activation is a recurrent theme in this study, as described below. *LINC00645* is expressed throughout the brain and maps to a large, coding gene poor region on chromosome 14. The closest is *NOVA1*, which is ~1Mb upstream and codes for a splicing co-factor that mediates the response to cellular and behavioral stress, and inflammatory cytokines [42,43]. *KAL1* is expressed throughout the brain and involved in neural migration [44]. *RTL1* is a retrotransposon-derived, paternally expressed gene that maps to a well-established imprinted locus on 14q32; it is expressed in the brain and has been found to be up-regulated by Neurogenin 2 during dorsal telencephalon development [45,46]. *PCDH7* is a non-clustered protocadherin expressed in the brain that was also found to be differentially expressed by Hill *et al*., one of 17 genes that overlapped between our respective studies, the others being *CRYAB*, *CCL2*, *SEZ6*, *STAC*, *MCM3*, *CDCA4*, *ARHGAP19*, *NCAPG2*, *CDKN1A*, *MUTYH*, *EIF4A2*, *ANTXR1*, *ATP1B1*, *ACTG2*, *EIF4G1*, *and PPP4R1* [8]. The overlap of the differentially expressed genes is statistically significant (p = 4.6e-9, hypergeometric test).

**Table 1:** Top 30 differentially expressed genes.

Gene	TPM (SC)	TPM (KD)	log2 FC	p-value	FDR
*TIMP1*	99.60	33.45	-1.57	1.61E-15	2.30E-11
*LINC00645*	9.39	2.12	-2.15	4.55E-14	3.25E-10
*IFITM3*	642.29	183.68	-1.81	3.02E-13	1.08E-09
*IFITM2*	316.95	101.49	-1.64	4.12E-13	1.17E-09
*KAL1*	25.13	10.69	-1.23	2.92E-11	6.93E-08
*RTL1*	22.79	5.36	-2.09	5.35E-10	9.53E-07
*PCDH7*	16.42	6.52	-1.33	8.76E-09	1.17E-05
*SPOCK1*	25.78	11.62	-1.15	2.43E-08	2.17E-05
*ZNF804A*	4.48	1.42	-1.66	9.43E-08	6.72E-05
*KCNF1*	13.92	4.21	-1.73	1.26E-07	8.18E-05
*THBS4*	17.42	8.41	-1.05	1.53E-07	8.51E-05
*CPNE4*	35.30	14.95	-1.24	2.03E-07	2.03E-07
*CRYAB*	7.79	0.88	-3.15	2.68E-07	2.68E-07
*LCNL1*	6.72	0.83	-3.01	6.57E-07	6.57E-07
*THBS2*	18.26	5.87	-1.64	2.12E-06	2.12E-06
*KIRREL2*	19.06	85.95	2.17	1.25E-13	5.93E-10
*ZIC3*	12.43	78.65	2.66	3.02E-10	6.15E-07
*MTFP1*	4.18	12.06	1.53	1.13E-09	1.79E-06
*PALM3*	7.31	20.94	1.52	9.19E-09	1.17E-05
*KIF4A*	17.03	46.56	1.45	9.85E-09	1.17E-05
*BAIAP2L1*	0.86	2.86	1.74	1.43E-08	1.57E-05
*CDON*	13.49	40.95	1.60	1.74E-08	1.77E-05
*KIAA0101*	47.25	98.89	1.07	1.87E-08	1.78E-05
*PLA2G4A*	2.61	9.06	1.79	2.59E-08	2.17E-05
*GINS3*	7.80	17.48	1.16	5.12E-08	4.06E-05
*OBSCN*	6.48	10.67	0.72	9.08E-08	6.72E-05
*COL9A1*	2.20	7.33	1.73	1.43E-07	8.51E-05
*CTSC*	10.71	34.50	1.69	1.51E-07	8.51E-05
*H1F0*	9.99	36.31	1.86	1.55E-07	8.51E-05
*NPTX1*	5.85	13.88	1.25	2.12E-07	1.07E-04

Top genes that decrease (- log2) or increase (+ log2) in ZNF804A KD cells. TPM (transcripts per million are the means from the 3 RNA-seq samples; SC (scrambled control); KD (knockdown). The FC (fold change) value is expressed as the log2 of the mean of KDs over the scrambled controls. FDR is the false discovery rate.

Among the four genes affected by *ZNF804A* in the Girgenti study, which was carried out in rat NPCs, one overlapped with our findings; the SZ and BD candidate *PDE4B* (increased in expression by overexpressing ZNF804A in rat NPCs, while it decreased following KD in our study—see S1 Table) [7].

Finally, two of the more interesting down-regulated genes are *IFITM2* and *IFITM3*, which decreased several fold (Table 1); they code for interferon-induced transmembrane proteins that play a role in interferon-signaling and the innate defense against influenza and other viruses [47–49]. This is particularly interesting from a SZ pathogenesis perspective, considering the clinical and epidemiological evidence pointing towards prenatal influenza as a risk factor in SZ, which will be described in more detail in the discussion [50–59].

One concern about the dramatic decrease in *IFITM2* and *IFITM3* expression in the KD lines is that it’s an artifact of lentiviral transduction; that is, could the reduced expression in the *ZNF804A* KD lines compared with the scrambled controls be due to differences in the expression of lentiviral sequences? However, in an analysis of *IFITM2* and *IFITM3* expression in this experiment and in 17 other KD and scrambled lines we have generated in NPCs, no correlation was observed between viral gene expression (inferred from RNA-seq reads mapped to vectors) and *IFITM2* and *IFITM3* RNA levels (correlation coefficients, -0.014 and 0.280, respectively).

In addition to these two interferon-related genes, a number of others that code for various proteins involved in cytokine signaling are significantly lower in the KD samples, including *IFIT3*, *IL17RC*, *IL17D*, *CCL2*, *CXCL16*, *IFNAR1*, *IL1RAP*, *IL6ST*, *C1QTNF5*, *TNFAIP3*, *TNFRSF21*, *TNFAIP8*, *TNFAIP8L1*, *TNFAIP2*, *C1QTNF6* and *TNFSF12* (S1 Table).

The most significantly up-regulated genes in the KD samples were *KIRREL2*, *ZIC3*, *MTFP1*, *PALM3*, and *KIF4A*. *KIRREL2* (Kin of IRRE-like 2), is a cell adhesion molecule that regulates activity-dependent axonal projections in the olfactory system of mice [60]. *ZIC3* is a member of a family of zinc-finger transcription factors that plays a role in regulating the proliferation and differentiation of neuronal progenitors in the medial forebrain and the cerebellum, retinoic acid signaling in the hindbrain, and the generation of mouse iPSCs [61–63]. Two other members of the ZIC family, *ZIC1* and *ZIC4* are also significantly up-regulated in the KD cells (S1 Table). *ZIC1* was also found to be differentially expressed in the Hill *et al ZNF804A* KD study, as noted above. *MTFP1* (mitochondrial fission process 1) is a nuclear gene that is a downstream target of the phosphatidylinositol 3-kinase signaling pathway, *PALM3* is a member of a multigene family involved in membrane dynamics, and is expressed in axons and dendritic spines; it is also up-regulated by LPS and may be involved in IL-1 signaling [64,65].

We validated the changes in gene expression for 12 genes including *ZNF804A*; all but one (*TCF4*) was confirmed (Fig 1). We chose to validate genes of interest in neuropsychiatric disorders, as well as several that overlapped with a study we carried out on neuronal aggregates derived from iPSCs exposed to heat shock [66]. *TCF4* expression decreased, as expected, but statistical significance was not achieved because of a large standard deviation.

### Pathway Analysis

Based on the function of the top differentially expressed genes, ZNF804A appears to have an effect on genes involved in immune responses. In order to evaluate this possibility more systematically, we assessed the 1815 differentially expressed genes that were nominally significant (p < 0.05) using Ingenuity Pathway Analysis (IPA). For genes that decreased in the *ZNF804A* KDs, the top disease pathways and biological functions were neuromuscular disease, dyskinesia, movement disorders and Huntington's Disease. Genes implicated in SZ and BD were the 5^th^ and 9^th^ most significant (Table 2; S2 Table for complete IPA analysis).

**Table 2:** Diseases and biological functions for ZNF804A down-regulated genes.

Diseases/Functions	p-value	Molecules
neuromuscular disease	1.56E-08	ADRA2A,AEBP1,AK5,ALDH6A1,APOE,ATP2B2,BRINP1,CCL2,CDS1,CHRM3,CNR1,CPNE5,CRYAB,CRYM,DGKB,ESRRG,FAS,FLRT2,GABRA2,GRIK2,HAP1,HCG22,IFNAR1,ITPKB,KCNA5,KCNAB1,KCND2,KCNIP1,MX1,NR4A2,PCDH7,PDE4B,PPARA,PPP3CA,PRNP,PSMB8,RXRG,SCARB2,SCN2A,SCN4B,SEZ6,SH3GL2,SLC1A1,SLC24A3,SPOCK3,ST8SIA4,TM2D1,TOMM20,TPD52
dyskinesia	1.31E-07	ADRA2A,AEBP1,ALDH6A1,APOE,ATP2B2,CDS1,CHRM3,CNR1,CPNE5,CRYAB,CRYM,DGKB,ESRRG,FLRT2,GABRA2,GRIK2,HAP1,ITPKB,KCNA5,KCNAB1,KCNIP1,PCDH7,PPARA,PPP3CA,PSMB8,RGS2,RXRG,SCARB2,SCN2A,SCN4B,SEZ6,SLC1A1,SPOCK3,TM2D1,TOMM20,TPD52
Movement Disorders	2.46E-07	ADRA2A,AEBP1,ALDH6A1,APOE,ATP2B2,BEAN1,CA5B,CDS1,CHRM3,CNR1,CPNE5,CRYAB,CRYM,DGKB,ESRRG,FAS,FLRT2,GABRA2,GRIK1,GRIK2,HAP1,ITPKB,KCNA5,KCNAB1,KCNC3,KCNIP1,KLHL1,NR4A2,PCDH7,PDE4B,PPARA,PPP3CA,PSMB8,RGS2,RXRG,SCARB2,SCN2A,SCN4B,SEZ6,SH3GL2,SLC1A1,SPOCK3,ST8SIA4,TM2D1,TOMM20,TPD52
Huntington's Disease	3.01E-07	AEBP1,ALDH6A1,APOE,ATP2B2,CDS1,CHRM3,CNR1,CPNE5,CRYAB,CRYM,DGKB,ESRRG,FLRT2,GABRA2,GRIK2,HAP1,ITPKB,KCNA5,KCNAB1,KCNIP1,PCDH7,PPARA,PPP3CA,PSMB8,RXRG,SCARB2,SCN2A,SCN4B,SEZ6,SLC1A1,SPOCK3,TM2D1,TOMM20,TPD52
Schizophrenia	5.13E-07	ADRA2A,ALDH3B1,ALDH5A1,APOE,ATP2B2,CALY,CCK,CHRM3,CNR1,CRYM,CXCL12,DAB1,EBF1,ELAVL4,GABRA2,GRIA4,GRID1,GRIK1,GRIK2,GRIK4,GRM5,HIST1H2BN,MCHR1,NELL1,PLA2G4C,PPARA,RXRG,SLC12A2,SLC12A5,SLC1A1,SLC6A1,SLIT3,SNRK,TF
disorder of basal ganglia	8.33E-07	ADRA2A,AEBP1,ALDH6A1,APOE,ATP2B2,CDS1,CHRM3,CNR1,CPNE5,CRYAB,CRYM,DGKB,ESRRG,FLRT2,GABRA2,GRIK2,HAP1,ITPKB,KCNA5,KCNAB1,KCNIP1,NR4A2,PCDH7,PDE4B,PDE8B,PPARA,PPP3CA,PSMB8,RGS2,RXRG,SCARB2,SCN2A,SCN4B,SEZ6,SH3GL2,SLC1A1,SPOCK3,ST8SIA4,TM2D1,TOMM20,TPD52
progressive motor neuropathy	3.73E-06	ADRA2A,AK5,ALDH5A1,APOE,BRINP1,CCL2,CDKN1A,CHRM3,CNR1,CRYAB,DAB1,FAS,GABRA2,HCG22,HRK,IFNAR1,KCND2,MX1,NR4A2,PDE4B,PLA2G4C,PRNP,SCN2A,SCN4B,SH3GL2,SLC1A1,SLC24A3,SPARC,ST8SIA4,SUSD1,TIMP1
epilepsy	1.12E-05	ALDH5A1,ARRDC3,ASAH1,CA5B,CDKN1A,CRYM,ERRFI1,GABRA2,GRIK1,KAL1,LINC00152,NR4A3,PRICKLE1,PRNP,RGS2,SCARB2,SCN2A,SCN4B,SLC6A1,TRIM9
bipolar disorder	3.32E-05	ADRA2A,ALDH5A1,APOE,CA5B,CCL2,CHRM3,GABRA2,GRIA2,GRID1,GRIK1,GRIK2,GRIK4,GRM5,MCHR1,PPP2R2C,PPP3CA,RABGAP1L,SCN2A,SCN4B,SLC1A1,TCF4
inflammatory demyelinating disease	6.41E-05	ADRA2A,AK5,BRINP1,CCL2,CHRM3,CNR1,CRYAB,FAS,HCG22,IFNAR1,KCND2,MX1,SLC24A3,TIMP1

This included several kainate ionotrophic receptors, AMPA and metabotropic glutamate receptor subtypes, and the GABA-A receptor subtype, *GABRA2* [67]. In addition, a decrease in cholecystokinin expression was detected in the KD neurons. CCK codes for a neuropeptide that’s expressed in a subset of GABAergic neurons; it has a role in mediating anxiety traits and panic disorder, and is differentially expressed in the entorhinal cortex in SZ [68–72].

Inspection of the list of down-regulated genes showed several others that are of interest in neuropsychiatric disorders, but were not called by IPA, including *TIMELESS*, *DISC1*, *PTEN*, *RANBP1*, *TCF4*, *NPAS3*, *PCDH9*, *NLGN4X*, *and CDH7* [73–84].

Consistent with the marked decrease we detected in *IFITM2* and *IFITM3*, IPA showed a significant enrichment for genes involved in inflammation and response to cytokines among the KD down-regulated genes. In the IPA “upstream regulators” analysis, in which IPA assesses the observed gene expression changes for transcriptional activators, the top 2, and 4 of the top 10 upstream regulators were members of the interferon family: IFNA2 (interferon alpha 2), IFNL1 (interferon lambda1), ISG15 (interferon stimulated gene 15), a ubiquitin-like modifier that is induced by type I interferons, and IFNG (interferon gamma) (Table 3). Similar results were obtained when the data were analyzed by Gene Ontology (GO): among the top GO terms were 3 out of 11 involved in interferon signaling (Table 4; S3 Table).

**Table 3:** Upstream Regulators for ZNF804A KD down-regulated genes.

Upstream Regulator	p-value	Target molecules in dataset
IFNA2	1.11E-08	C19orf66,FAS,HERC6,IFI35,IFI44L,IFI6,IFIT3,IFITM3,IRF9,ISG20,LGALS3BP,MX1,PARP12
IFNL1	2.44E-08	C19orf66,HERC6,IFI35,IFI44L,IFI6,IFIT3,IFITM3,IRF9,ISG20,LGALS3BP,MX1
MAPK1	4.07E-06	CDKN1A,DEPTOR,IFI16,IFI35,IFI6,IFIT3,IFITM3,IRF9,ISG20,LGALS3BP,MVP,PARP12,PSMB8,SPOCK1
USP18	1.02E-05	IFI6,IFITM3,IRF9,MX1
ISG15	3.09E-05	IFI6,IFITM3,MX1
EIF2AK2	8.26E-05	IFI35,IFI6,IFITM2,ISG20,LGALS3BP,PARP12,PARP9
IFNG	3.16E-04	CCL2,CDKN1A,CXCL16,FAS,HERC6,HRK,IFI44L,IFI6,IFIT3,IGFBP4,IRF9,ISG20,MCHR1,MX1,PSMB8,TNFSF12
PKC(s)	3.39E-04	CCL2,IGF2,NR4A3,PPARA,RGS2
Smad1/5/8	3.96E-04	CDKN1A,CDKN2A
LIN9	3.96E-04	CDKN1A,CDKN2A

**Table 4:** Gene Ontology (GO) for genes down-regulated following *ZNF804A* KD.

GO term	Biological process	# DEGs	p-value	FDR
GO:0003008	system process	72	1.43E-09	5.03E-06
GO:0044707	single-multicellular organism process	169	7.41E-09	1.31E-05
GO:0007268	synaptic transmission	41	1.53E-08	1.80E-05
GO:0032501	multicellular organismal process	171	2.40E-08	2.12E-05
GO:0050877	neurological system process	56	4.61E-08	3.25E-05
GO:0035637	multicellular organismal signaling	44	6.50E-08	3.82E-05
GO:0019226	transmission of nerve impulse	43	8.32E-08	4.20E-05
GO:0048731	system development	119	3.70E-07	1.63E-04
GO:0071357	cellular response to type I interferon	10	4.87E-07	1.72E-04
GO:0060337	type I interferon signaling pathway	10	4.87E-07	1.72E-04
GO:0034340	response to type I interferon	10	6.13E-07	1.97E-04
GO:0007267	cell-cell signaling	48	6.94E-07	2.04E-04
GO:0023052	signaling	150	9.25E-07	2.33E-04
GO:0044700	single organism signaling	150	9.25E-07	2.33E-04
GO:0007154	cell communication	152	1.46E-06	3.43E-04
GO:0051239	regulation of multicellular organismal process	69	7.57E-06	0.0017
GO:0007610	behavior	29	9.39E-06	0.002

**Fig 2:**
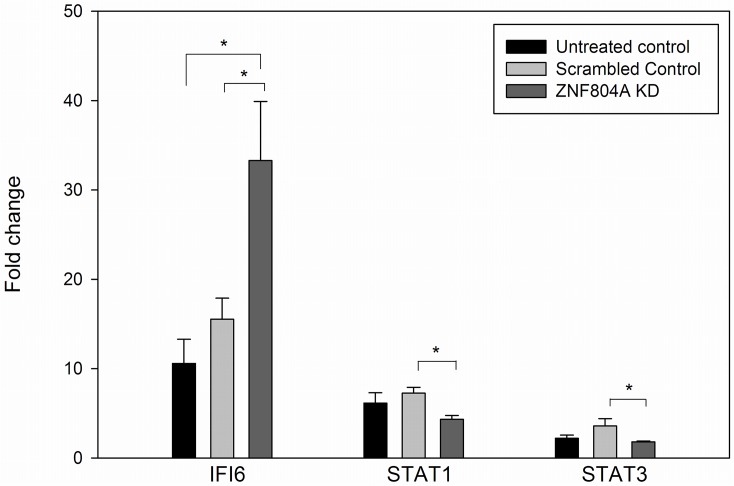
Induction of interferon-responsive genes. NPCs were treated with IFNA2 (1mg/ml) for 4 hours, after which RNA was isolated and analyzed by qPCR. *IFI6* and *STAT1* increased with treatment, while *STAT3* decreased. Fold changes were calculated as described in the methods section. Significant differences in the fold change were found in the comparison between the scrambled control and the *ZNF804A* KD line for all three genes using a Student’s t-test (all results two-tailed): *IFI6*, p = 0.03; *STAT1*, p = 0.004; *STAT3*, p = 0.05. No other significant differences were found for the other comparison, except for untreated control vs *ZNF804A* KD (p = 0.01). The bar graph represents the mean of 6 determinations (biological duplicates analyzed in triplicate) +/- standard error.

The IPA findings suggest that interferon signaling will be affected by ZNF804A. This is supported by an analysis of IFNA2-mediated gene expression carried out in NPCs from an untreated control, a scrambled control, and a *ZNF804A* KD, as described in the methods section. Following treatment with IFNA2, *IFI6* and *STAT1* gene expression significantly increased, while STAT3 decreased significantly (~2–10 fold changes in expression compared with controls; all p-values <0.05, not shown). As seen in Fig 2, there was a statistically significant increase in the fold induction of *IFI6* in the KD samples compared with both the untreated control NPC line used for the shRNA experiment, and the scrambled control KD (~2-fold more robust induction with INFA2; p = 0.01 and 0.03, respectively, two-tailed Student’s t-test). By contrast, no significant differences were found when the untreated control and the scrambled control were compared. Significant differences were also found in the *ZNF804A* KD for STAT1 and STAT3 in comparison with the scrambled control (p = 0.004; 0.05, respectively). However, for these two genes, the induction (*STAT1*) or decrease (*STAT3*) that occurs following INFA2 treatment was attenuated compared with the scrambled control. These findings support that idea that INFA2 signaling is affected by *ZNF804A*.

**Fig 3:**
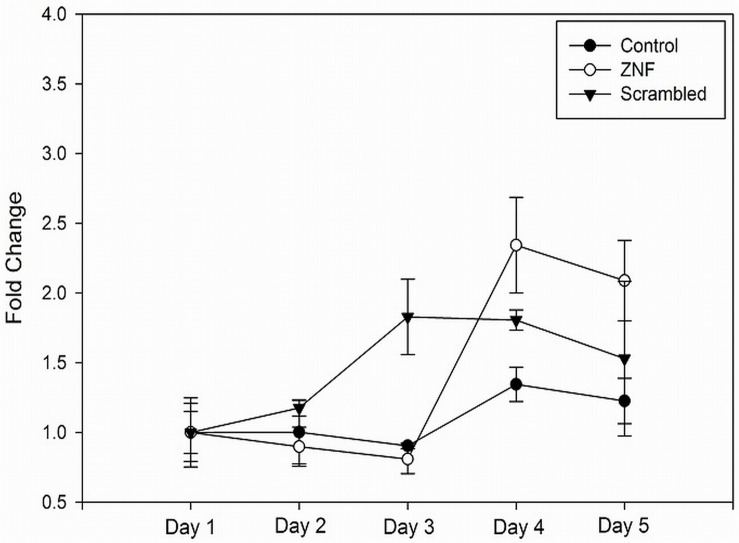
Proliferation assay. NPCs were plated at 50,000 cells/well and analyzed using a Vybrant MTT cell proliferation assay kit, as described in the methods section. The samples analyzed included a *ZNF804A* KD line, a scrambled control line, and an un-processed control (no virus transduction or puromycin selection) generated from an iPSC clone derived from the same subject used for the *ZNF804A* KD. Samples were analyzed in triplicate and the data are presented as means, +/- standard error. Differences were not statistically significant, although a trend towards significance was found in the comparison between the KD and un-processed control on days 4 and 5 (Student’s t-test, two tailed, p = 0.052 and 0.056, respectively).

For the up-regulated genes, the top disease pathways and biological functions were all related to the mitotic cell cycle, cellular assembly, DNA replication, and cancer with very low p-values ranging from E-19 to E-31 (S4 Table). Similar results were obtained when the enriched GO terms were determined (Table 5 and S5 Table for complete list). The findings suggest that knocking down *ZNF804A* affects proliferation. To test this idea, a proliferation assay was carried out in NPCs. After a three day lag period, the *ZNF804A* KD line showed a 30–37% increase in proliferative capacity compared with a scrambled control on days 4 and 5. However, differences were not statistically significant (Fig 3). The *ZNF801A* KD line also showed an increase in proliferation compared with the untreated control (wild type line; parent line, but not transduced with an shRNA vector), which almost reached statistical significance (Student’s t-test, two tailed, p = 0.052 and 0.056 for days 4 and 5, respectively).

**Table 5:** Gene Ontology (GO) for ZNF804A KD up-regulated genes.

GO_term	Biological process	# of DEGs	p-value	FDR
GO:0000278	mitotic cell cycle	162	5.07E-65	2.05E-61
GO:0007049	cell cycle	203	2.29E-59	4.63E-56
GO:0022402	cell cycle process	176	3.14E-58	4.24E-55
GO:0000280	nuclear division	96	2.19E-50	1.78E-47
GO:0007067	mitosis	96	2.19E-50	1.78E-47
GO:0048285	organelle fission	98	5.92E-49	4.00E-46
GO:0051301	cell division	115	1.88E-46	1.09E-43
GO:1902589	single-organism organelle organization	175	1.17E-37	5.92E-35
GO:0006259	DNA metabolic process	121	1.15E-34	5.19E-32
GO:0007059	chromosome segregation	51	3.12E-32	1.26E-29

The marginal, albeit statistical insignificant increase in proliferation could suggest that some of the changes in the expression of genes implicated in neuropsychiatric disorders and in cytokine-signaling found in day 14 neurons are secondary to an effect on proliferation and maturation from NPCs into neurons. However, there was no consistent difference in expression of NPC markers; *SOX2*, *VIM* (vimentin) and *PAX6* expression levels did not differ between the KD and scrambled control, although *NES* (nestin) expression increased by ~40% (S1 Table). It should be noted, however, that *IFITM2* and *IFITM3*, are not differentially expressed during the transition from NPCs to neurons [30]. A decrease in *IFITM2* and *IFITM3* expression independent of differentiation status was confirmed by qPCR carried out on KD and scrambled control NPCs (p = 0.04 and 0.001, respectively; not shown). Thus, the differentially expressed genes we detected in the KD experiment in day 14 neurons do not appear to be an artifact of differential proliferation or differentiation status.

## Discussion

SZ, ASD and BD, and other neuropsychiatric disorders are genetically heterogeneous complex genetic traits that will make it difficult to translate genetic findings into novel therapeutics. One way to address heterogeneity is to find common pathways onto which different candidate genes converge. This is particularly feasible for genes that code for transcription factors or chromatin remodeling proteins that could potentially have an impact on shared target genes or pathways. A convergence of target genes is suggested by our finding that the expression of a number of SZ and BD candidates was affected by *ZNF804A* KD, as noted above.

The utility of identifying common molecular and genetic targets could also be applied when considering the role of environmental factors in the development of SZ and ASD. Although these disorders are highly heritable, environmental factors play a role as well; maternal immune activation (MIA) caused by infectious diseases or autoimmune phenomena, maternal nutritional deprivation, obstetrical complications, oxidative stress, anoxic stress, paternal age and environmental toxins have all been implicated [85–95].

Although these environmental factors could have effects on neurogenesis and brain development that are independent of known genetic risk factors, there is experimental support for their convergence in some circumstances. We have recently found, for example, that hyperthermia (heat shock) markedly alters the expression of several SZ, BD and ASD candidate genes, including *ZNF804A*, *ARNT2* and *SMARCA2*, in a model of the human fetal telencephalon derived from iPSCs [66,96]. In addition, in mouse models, gene x environment interactions involving the SZ, BD, and ASD candidate genes *DISC1*, *NRG1*, *RGS4*, and *AKT1* have been described [97–102].

One major finding in this study—altered expression of genes involved in cytokine signaling following *ZNF804A* KD—is consistent with these observations, and suggests that allelic variants in the gene interact with cytokine signaling pathways in some manner leading to an increased risk of disease. One of the more interesting cytokine-related findings, a decrease in *IFITM2* and *IFITM3* gene expression, suggests another possibility; an impact on viral pathogens that have cytopathic effects on neurons. *IFITM2* and *IFITM3* code for interferon-induced transmembrane proteins, which are viral restriction factors that play a role in protecting cells against the entry of influenza and other viruses, as well as other aspects of interferon-signaling [47–49]. This is of interest because of previous studies suggesting that maternal exposure to influenza and other viruses increases SZ risk in offspring [50–56]. Thus, a decrease in *IFITM*2 and *IFITM3* expression, for example due to reduced *ZNF804A* expression or function, could increase the cytopathogenic effects of some viruses. Investigators have reported an alteration in *IFITM2* and *IFITM3* gene expression in various brain regions in SZ and ASD autopsy samples; however, expression is increased [57,103–108]. The opposite effects on expression in our *ZNF804A* KD neurons and the autopsy studies could be due to differences related to neuronal maturation or stage of brain development, as well the obvious differences in the choice of tissue: live cells vs autopsy samples. In addition, *IFITM2* and *IFITM3* are many fold more highly expressed in non-neuronal cells in the brain, such as endothelium and microglia, so differential expression in the brain vs pure neuronal cultures in could be due opposite effects of these genes in neurons compared with other cell types [109].

In addition to *IFITM2* and *IFITM3* **,** a number of other genes involved in interferon signaling were differentially expressed, as described in the results section. This too is consistent with a number of findings in humans and animal models. For example, an increase in INF-γ levels was found in the CSF of patients with SZ and in the plasma of patients with first episode psychosis [110–111]. And, an alteration in the ratio of IFN-γ to various interleukins was found to be significantly decreased in SZ, consistent with a Th2 shift [112]. Finally, an increase in serum IFN-γ was found in patients with 22q11.2 del who experienced ASD-like behaviors [113].

As for other cytokines, several genes involved in interleukin and TNF signaling were also found to be differentially expressed in the *ZNF804A* KD samples, as noted above. These could be meaningful considering that fact that IL-1β, IL-6 and TNF-α mediate the effects of MIA on brain development and behavior in animal models, and altered expression has been found in the brain and peripheral blood of patients with SZ and ASD [114–117]. And interestingly, differences in IL-17 expression have been found in SZ and ASD compared with controls in first episode, medication naïve SZ patients, and animal models [118–124].

Two other down-regulated genes related to immune function that we detected were *CCL2* and *CXCL16*; *CCL2* was also significantly reduced in the Hill *et al*. study, as noted in the results section [8]. *CCL2* codes for a chemokine expressed in neurons, microglia, and astrocytes that is induced by viral infection, head trauma, and cytokines [40,125–130]. A number of studies show that plasma CCL2 levels differ in SZ, ASD, BD and major depression compared to controls [120,131–136]. Although chemokines are primarily known for their role in innate immunity, in particular chemotaxis, they are increasingly being recognized for their non-immune effects in the brain (neurogenesis, neuroprotection, dopamine transmission, migration of NPCs, and controlling blood–brain barrier permeability) [137–141]**.**

*CXCL16* codes for a chemokine that is primarily expressed in lymphoid tissue, but also appears to have a protective effect on excitotoxic cell death in the brain and is induced in a mouse model of multiple sclerosis [142,143]. In addition, SNPs in the gene were found to be associated with SZ in a combined GWAS/pathway analysis [144].

Another differentially expressed gene worth noting in the general context of inflammatory responses is *CRYAB*, which decreased in the KD cells, similar to the Hill *et al*. study [8]. *CRYAB* codes for heat shock inducible chaperone that suppresses apoptosis and neuroinflammation [145,146]. It was one of the top genes induced by heat shock in human fetal telencephalon-like structures we differentiated from iPSCs [66]. This suggests that reduced expression caused by a decrease in ZNF804A could make differentiating neurons more vulnerable to infectious disease, autoimmune attack or cellular stress.

Overall, our results suggest that *ZNF804A* modulates the expression of genes that regulate cytokine and chemokine signaling pathways in differentiating neurons, perhaps affecting their response to immune activation and/or viral infection. This hypothesis is also supported by immunological studies and replicated GWAS findings, in which association signals in the MHC locus have been found, suggesting an infectious disease and/or autoimmune process in subgroups of SZ and ASD patients [147–158]. However, non-immune effects of MHC antigens on brain development and neuronal function is a possible explanation for the association [159–161].

Finally, it should be noted that an effect of *ZNF804A* on cytokine signaling could also help explain the development of metabolic syndrome, which occurs as a polymorphic trait in many patients treated with psychotrophic medications (and some untreated patients as well); metabolic syndrome is mediated by immune cytokines [133,162–167]. It should also be noted that there are four binding sites for EGR (early growth response) transcription factors in the *ZNF804A* promoter region. EGR transcription factors play a role in synaptic plasticity and behavior, and are activated by pro-inflammatory cytokines, providing another potential connection between immune activation and *ZNF804A* expression, and suggesting the possibility of feedback interactions [168–172].

These findings, while interesting, must be viewed cautiously, however. Although we did not detect a correlation between expression of lentiviral sequences and *IFITM2* and *IFITM3* expression, it is still possible that the changes we detected in cytokine signaling genes are an artifact of viral transduction that cannot be explained simply by analyzing vector transcripts. Consequently, validation using a gene knockout approach with CRISPR or zinc finger nucleases, rather than shRNA mediated gene KD will be extremely valuable to confirm our findings, as well as performing these analyses using additional iPSC lines to account for genetic background differences.

## Supporting Information

S1 Fig Germ line markers are expressed in the iPSC line used in this experiment: AFP (alpha-fetoprotein; endoderm), TUJ1 (TUBB3; Neuron-specific class III beta-tubulin) (ectoderm), Desmin (mesoderm). In addition, the iPSC line expresses the pluripotency markers Tra 1–60 and Tra-81, and has a normal karyotype.
(JPG)

S1 Table Entire list of RNA-seq data from 3 scrambled controls and 3 *ZNF804A* KDs arranged by lowest FDR. All genes showing genome-wide significance (FDR; q-value <0.05) are highlighted in bold type.
(XLSX)

S2 Table Ingenuity Pathway Analysis (IPA) for ZNF804A down-regulated genes. (XLS)

S3 Table Gene Ontology (GO) for ZNF804A down-regulated genes. (XLSX)

S4 Table Ingenuity Pathway Analysis (IPA) for ZNF804A up-regulated genes. (XLS)

S5 Table Gene Ontology (GO) for ZNF804A up-regulated genes. (XLSX)

S1 Text Comprehensive description of methods, PCR primers and antibodies. (DOCX)
