# Supplementary figures and images for: ZNF804A Transcriptional Networks in Differentiating Neurons Derived from Induced Pluripotent Stem Cells of Human Origin
Source: PLoS One. 2015 Apr 23;10(4):e0124597. doi: 10.1371/journal.pone.0124597 (PMC4408091; doi:10.1371/journal.pone.0124597)

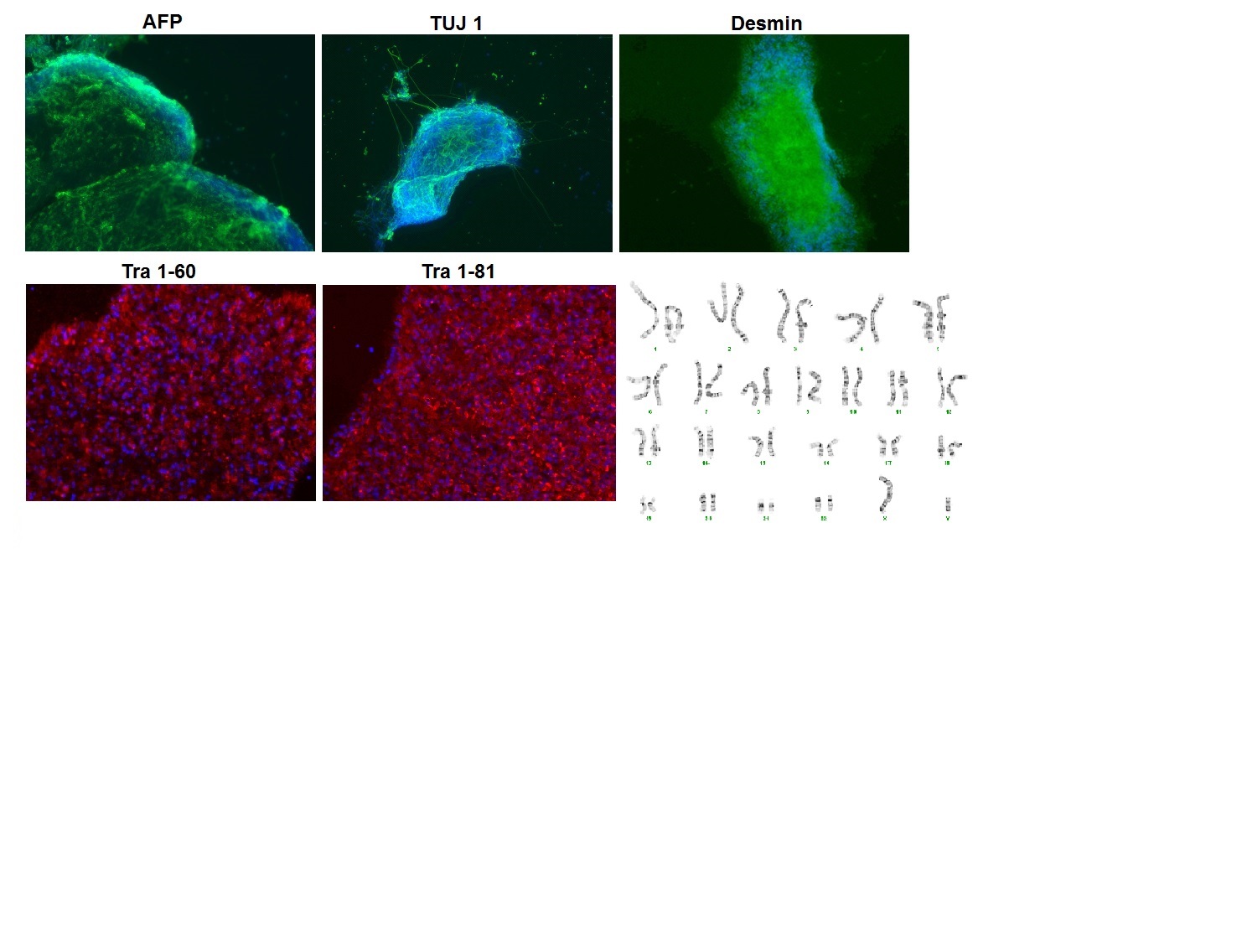

Supplement: S1 Fig — In addition, the iPSC line expresses the pluripotency markers Tra 1–60 and Tra-81, and has a normal karyotype. (JPG) [file pone.0124597.s001.jpg]
